# High abundance of virulence gene homologues in marine bacteria

**DOI:** 10.1111/j.1462-2920.2008.01861.x

**Published:** 2009-06

**Authors:** Olof P Persson, Jarone Pinhassi, Lasse Riemann, Britt-Inger Marklund, Mikael Rhen, Staffan Normark, José M González, Åke Hagström

**Affiliations:** 1 Marine Microbiology, Department of Natural Sciences, University of KalmarSE-39182 Kalmar, Sweden; 2 Department of Microbiology, Tumor and Cell Biology, Karolinska InstitutetSE-17771 Stockholm, Sweden; 3 Department of Microbiology and Cell Biology, University of La LagunaES-38206 La Laguna, Tenerife, Spain

## Abstract

Marine bacteria can cause harm to single-celled and multicellular eukaryotes. However, relatively little is known about the underlying genetic basis for marine bacterial interactions with higher organisms. We examined whole-genome sequences from a large number of marine bacteria for the prevalence of homologues to virulence genes and pathogenicity islands known from bacteria that are pathogenic to terrestrial animals and plants. As many as 60 out of 119 genomes of marine bacteria, with no known association to infectious disease, harboured genes of virulence-associated types III, IV, V and VI protein secretion systems. Type III secretion was relatively uncommon, while type IV was widespread among alphaproteobacteria (particularly among roseobacters) and type VI was primarily found among gammaproteobacteria. Other examples included homologues of the *Yersinia* murine toxin and a phage-related ‘antifeeding’ island. Analysis of the Global Ocean Sampling metagenomic data indicated that virulence genes were present in up to 8% of the planktonic bacteria, with highest values in productive waters. From a marine ecology perspective, expression of these widely distributed genes would indicate that some bacteria infect or even consume live cells, that is, generate a previously unrecognized flow of organic matter and nutrients directly from eukaryotes to bacteria.

## Introduction

Pathogenic bacteria have a profound impact on plants, animals and humans. Thus, understanding the origin and distribution of genes that convey pathogenicity is a central issue. These virulence genes represent virulence factors that are used by the bacteria for attachment to and degradation of eukaryotic cells. In particular, bacterial protein secretion systems are crucial for pathogenicity as they allow export of virulence factors. The molecular maps that describe the interaction between bacterial pathogens and their hosts have largely been generated through research on infectious disease and its causative agents. The extent to which homologues to such virulence factors are present in environmental bacteria is less known (Chakraborty *et al*., 2000; Dziejman *et al*., 2005; Bingle *et al*., 2008). Interestingly, such genes are widespread in soil bacteria (Casadevall, 2006), where they supposedly allow bacteria to interact with eukaryotes. Analogously Moran *et al*. (2007) suggested that aquatic heterotrophic prokaryotes could prosper at the expense of marine eukaryotes using the same principles as known pathogens.

The typical genome composition of a pathogenic bacterium consists of a core genome shared with related commensals, yet augmented with varying genetic elements that appear to fall into a few strategic categories (Hochhut *et al*., 2005; Gal-Mor and Finlay, 2006), for example, coding for the capacity of adhesion to host surfaces through the expression of adhesins to enable colonization, for molecules that interfere with intrinsic host cell functions, and for the ability to deliver such molecules (effector proteins) upon contact with their cognate host target. Often, the virulence genes are contained on extended genetic continuums termed pathogenicity islands (PAIs, see Schmidt and Hensel, 2004 for review). The PAIs vary in size from a few kilobases to large chromosomal regions, and their base composition and codon usage often differ from the host genome. They are frequently integrated in proximity to tRNA or insertion sequences, or are flanked by direct repeats. This implies that PAIs can move between different bacterial species through horizontal gene transfer (Schmidt and Hensel, 2004).

The original sources of virulence genes and PAIs, however, remain to be elucidated. It has been suggested that virulence genes in soil bacteria represent a reservoir for virulence genes found in human, animal, plant and insect pathogens (Casadevall, 2006). Similarly, one could expect that the long evolutionary history of marine bacteria, preceding terrestrial life by almost three billion years, would make them an interesting target in the search for virulence genes. Marine bacterioplankton experience a dynamic environment and show diverse interactions with other plankton organisms. For example, there are numerous examples of marine bacteria causing harm to algae (see Mayali and Azam, 2004 for a review). Recent comparative genome analyses of three members of the marine *Roseobacter* clade by Moran and colleagues (2007) have provided important insights into the lifestyles of bacteria in this clade. Similar to known pathogens, the *Roseobacter* clade genomes consist of a core set of genes and a much larger number of additional genes present in only a single or few genomes. Interestingly, a number of virulence genes were found in the *Roseobacter* genomes and these were suggested to permit roseobacters to ‘directly capture organic matter from eukaryotic cells’ (Moran *et al*., 2007). The authors in this case suggest a ‘mix-and-match’ genome arrangement that would allow adaptation to a wide diversity of ecological niches.

In this study, we carried out an extensive *in silico* analysis to examine the prevalence of homologues of virulence genes and PAIs, known from bacteria that are pathogenic to terrestrial animals and plants, in whole-genome sequenced marine bacteria. Further, the frequency of these genes in the ocean was estimated by analysis of the metagenome data set from the Global Ocean Sampling expedition (Rusch *et al*., 2007).

## Results and discussion

The search and identification of virulence gene homologues in marine bacteria were based on three approaches: (i) blast analysis to generate amino acid sequence similarity values, (ii) classification of proteins within PFAM, TIGRFAM or COG domain structure libraries and (iii) gene arrangement, that is, conservation of operon structure of defined PAIs (see *Methods* section for details). We found virulence genes in 60 out of 119 analysed bacteria (Table 1, for complete list of genomes included in the study see Table S1) and, in addition, we found numerous other genes and operons encoding putative adhesins, surface structures and protein secretion, and chemotactic sensory systems, which may also play important roles in marine bacterial behaviour.

**Table 1:** Secretion systems and PAIs in genome sequenced marine bacteria.

Organism	Tax	T3SS	T4SS	T5SS	T6SS	Antifeeding island	Novel PAI
*Leeuwenhoekiella blandensis* MED217	Bact	–	**–**	**–**	**–**	**1**	**–**
*Microscilla marina* ATCC 23134	Bact	–	**–**	**–**	**–**	**1**	**–**
*Algoriphagus* sp. PR1	Bact	–	**–**	**–**	**–**	**1**	**–**
*Kordia algicida* OT-1	Bact	–	**–**	**–**	**–**	**1**	**–**
*Blastopirellula marina* DSM3645	Pla	–	**–**	**–**	**1**^a^	**–**	**–**
Alphaproteobacterium BAL199	α	–	**–**	**–**	**2**	**–**	**–**
*Erythrobacter litoralis* HTCC2594	α	–	**1**^b^	**1**^c^	**–**	**1**	**–**
*Erythrobacter* sp. NAP1	α	–	**1**^b^	**2**^d^	**–**	**–**	**–**
*Erythrobacter* sp. SD-21	α	–	**1**	**–**	**–**	**–**	**–**
*Sphingomonas* sp. SKA58	α	**1**	**1**	**–**	**–**	**–**	**–**
*Brevundimonas* sp. BAL3	α	–	**1**	**1**	**–**	**–**	**–**
*Nitrobacter* sp. Nb311A	α	–	**–**	**–**	**–**	**1**	**–**
*Fulvimarina pelagi* HTCC2506	α	–	**1**	**–**	**–**	**–**	**–**
*Stappia aggregata* IAM 12614	α	**1**	**1**	**1**	**1**	**–**	**–**
*Pseudovibrio* sp. JE062	α	**1**	**–**	**–**	**2**	**–**	**–**
*Roseobacter* sp. SK209-2-6	α	–	**–**	**–**	**1**	**–**	**–**
*Oceanicola batsensis* HTCC2597	α	–	**2**	**1**	**1**	**–**	**–**
Rhodobacterales bacterium HTCC2654	α	–	**3**	**–**	**–**	**1**	**–**
*Roseovarius nubinhibens* ISM	α	–	**1**	**–**	**–**	**–**	**–**
*Roseovarius* sp. TM1035	α	–	**1**	**–**	**–**	**–**	**–**
*Roseovarius* sp. 217	α	–	**3**	**1**^d^	**1**	**–**	**–**
*Roseobacter* sp. MED193	α	–	**2**	**–**	**1**	**–**	**–**
*Roseobacter litoralis* Och 149	α	–	**–**	**–**	**1**	**–**	**–**
*Phaeobacter gallaeciensis* BS107	α	–	**1**	**–**	**–**	**–**	**–**
*Roseovarius* sp. HTCC2601	α	–	**–**	**1**	**2**	**–**	**–**
*Roseobacter* sp. CCS2	α	–	**–**	**1**	**–**	**–**	**–**
Rhodobacterales bacterium HTCC2083	α	–	**–**	**–**	**1**	**–**	**–**
*Sagittula stellata* E-37	α	–	**1**	**–**	**1**	**–**	**–**
*Oceanibulbus indolifex* HEL-45	α	–	**3**	**–**	**–**	**–**	**–**
*Sulfitobacter* sp. NAS-14.1	α	–	**2**	**–**	**–**	**–**	**–**
*Roseobacter* sp. GAI101	α	–	**–**	**–**	**1**	**–**	**–**
Rhodobacterales bacterium Y4I	α	–	**–**	**–**	**1**	**–**	**–**
Rhodobacterales KLH11	α	–	**1**	**3**	**–**	**1**	**–**
*Pelagibacter ubique* HTCC1062	α	–	**–**	**–**	**–**	**–**	**–**
Methylophilales bacterium HTCC2181	β	–	**–**	**1**^d^	**–**	**–**	**–**
*Betaproteobacteria* sp. KB13	β	–	**–**	**1**	**–**	**–**	**–**
*Marinomonas* sp. MED121	γ	–	**–**	**–**	**2**	**–**	**–**
*Marinobacter* sp. DG893	γ	–	**–**	**–**	**1**	**–**	**–**
*Marinobacter* sp. ELB17	γ	–	**–**	**–**	**1**	**–**	**–**
*Methylophaga* sp. DMS010	γ	–	**–**	**1**	**–**	**–**	**–**
*Alcanivorax* sp. DG881	γ	–	**–**	**1**	**1**	**–**	**–**
*Limnobacter* sp. MED105	γ	**1**	**–**	**3**	**1**	**–**	**–**
*Moritella* sp. PE36	γ	–	**–**	**–**	**2**	**–**	**–**
*Nitrococcus mobilis* Nb-231	γ	–	**–**	**–**	**1**	**1**	**–**
*Oceanobacter* sp. RED65	γ	–	**–**	**2**	**1**	**–**	**–**
Gammaproteobacterium HTCC2080	γ	–	**–**	**–**	**1**	**–**	**–**
Alteromonadales TW-7	γ	–	**–**	**–**	**1**	**–**	**–**
*Pseudoalteromonas tunicata* D2	γ	–	**–**	**–**	**1**^e^	**1**	**–**
*Psychromonas* sp. CNPT3	γ	–	**–**	**–**	**1**	**–**	**–**
*Reinekea blandensis* MED297	γ	–	**–**	**–**	**1**	**–**	**–**
*Stenotrophomonas* sp. SKA14	γ	–	**–**	**1**^d^	**1**	**–**	**–**
*Photobacterium* sp. SKA34	γ	–	**–**	**–**	**1**	**–**	**1**
*Vibrio angustum* S14	γ	–	**–**	**–**	**1**	**–**	**1**
*Vibrio fischeri* MJ11	γ	–	**–**	**–**	**2**	**–**	**–**
*Vibrio shilonii* AK1	γ	–	**–**	**–**	**1**	**–**	**–**
*Vibrio alginolyticus* 12G01	γ	**1**	**–**	**–**	**2**	**–**	**–**
*Vibrio* sp. MED222	γ	–	**–**	**–**	**1**	**–**	**–**
*Vibrio splendidus* 12B01	γ	–	**–**	**–**	**1**	**–**	**–**
Vibrionales bacterium SWAT-3	γ	–	**–**	**–**	**3**	**–**	**–**
*Vibrio campbellii* AND4	γ	**1**	**–**	**–**	**1**	**1**	**–**
*Plesiocystis pacifica* SIR-1	δ	–	**–**	**–**	**1**	**1**	**–**

a Lacks ImcF.

b Lacks VirB8.

c Lacks POTRA.

d Lacks ShlB.

e Lacks ImcF and ImpA.

The presence of the corresponding genomic island is indicated by digits denoting the number of occurrences; ‘–’ denotes not detected.

Bact, *Bacteroidetes*; α, *Alphaproteobacteria*; β, *Betaproteobacteria*; γ, *Gammaproteobacteria*; Pla, *Planctomycetes*.

### Protein secretion systems

Most pathogenic bacteria need to produce and export virulence factors, enzymes or toxins, in order to invade a host cell. To secrete nucleic acids or proteins through the outer membrane, Gram-negative bacteria apply specialized translocation systems. Predicted homologues to genes of known virulence-associated types III, IV, V and VI secretion systems were found in a large portion of the analysed marine bacteria (Table 1).

The type III secretion system (T3SS), or ‘injectisome’, exports proteins from the bacterial cytoplasm into eukaryotic cells, and helps mediating the interaction of bacterial symbionts or pathogens with their host (Cornelis, 2006). The exported effector molecules can, for example, enable tissue invasion and/or inhibition of host defences (Mota and Cornelis, 2005). We found T3SSs in six marine bacteria (Table 1), where putative T3SS genes were located in genome regions encompassing at least 17–43 genes (*Sphingomonas* sp. SKA58 and *Vibrio alginolyticus* 12G01 respectively). In type III secretion, specific ATPases are required for the actual secretion event; and the marine bacterial T3SS genome regions contained type III secretion ATPases with amino acid sequence similarities between 44% and 82% to the corresponding ATPase in *Yersinia pestis* CO92. A T3SS similar to that found in *Y. pestis* CO92 was found in *V. alginolyticus* 12G01, in accordance with previous reports (Park *et al*., 2004). The T3SSs are found in a wide variety of gammaproteobacteria, but appear less common in alphaproteobacteria (Pallen *et al*., 2005; Troisfontaines and Cornelis, 2005). We found putative T3SSs in three alphaproteobacteria, where previously not described. One of these systems, found associated with a large plasmid in *Sphingomonas* sp. SKA58, apparently shared features typical for both T3SSs and the structurally related flagellar motor.

The type IV secretion system (T4SS) is involved in DNA and/or protein transfer into prokaryotic and eukaryotic cells, and accounts for translocation of virulence factors in a number of bacterial pathogens (Cascales and Christie, 2003). Studies of the model plant pathogen *Agrobacterium tumefaciens* (alphaproteobacteria) have provided molecular detail on the VirB/D4 T4SS (Christie *et al*., 2005). Moran and colleagues (2007) found putative T4SSs in seven genome-sequenced roseobacters (some of which were also included in the present study). Among the diverse marine bacteria studied here, putative T4SSs of the VirB/D4 type were exclusively found in alphaproteobacteria (18 genomes), with one to three *vir* gene operons per genome (Table 1). This secretion system was amply distributed among members of the *Roseobacter* clade, but was also present in members of the families *Erythrobacteraceae* and *Caulobacteraceae*.

The two-partner secretion (TPS) system, a variant of the type V secretion system (T5SS), typically consists of a secreted TpsA protein (generally an adhesin) and a TpsB protein that facilitates the secretion of TpsA (Jacob-Dubuisson *et al*., 2001). In *Bordetella pertussis*, the causative agent of whooping cough, the TPS system secretes the filamentous haemagglutinin, which mediates adhesion to ciliated cells of the upper respiratory tract (Clantin *et al*., 2004). Other examples of TPS-mediated adhesion include cell–root interaction of *Pseudomonas putida* KT2440 (Molina *et al*., 2006) and the binding to red-blood cells in *Proteus mirabilis* (Bailey *et al*., 2005). Operons similar to TPS were found in 16 marine bacteria (Table 1). In *Oceanobacter* sp. RED65 we found two copies of TPS operons. The first TPS operon encoded two different putative haemagglutinins/adhesines, one having a similarity of 41% conserved amino acids to the filamentous haemagglutinin of *B. pertussis*. The second TPS operon encoded a protein with 38% similarity to HmwA filamentous haemagglutinin/adhesin of the human pathogen *Haemophilus influenzae*. The predicted size of the three variants of the putative excreted virulence factors in strain RED65 ranged from 80.3 to 306.1 kDa.

**Fig. 1:**
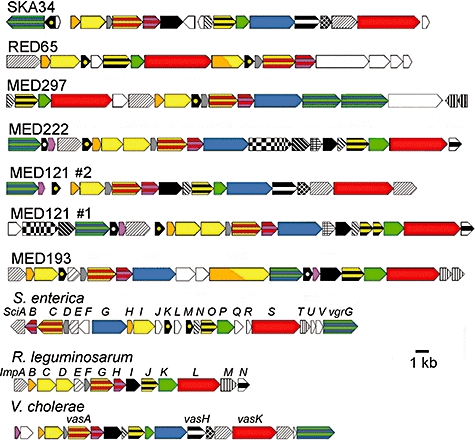
Gene organization of T6SSs in five representatives of the marine bacteria compared with those of *Salmonella enterica* subsp. I, *Rhizobium leguminosarum* biovar *trifolii*, and *Vibrio cholerae* O1 biovar eltor str. N16961. Predicted gene homologues are depicted with the same colours and patterns. The following genetic regions are shown: *Photobacterium* sp. SKA34 (SKA34_05615 to SKA34_05715), *Oceanobacter* sp. RED65 (RED65_00815 to RED65_00895), *Reinekea* sp. MED297 (MED297_18458 to MED297_18883), *Vibrio* sp. MED222 (MED222_13880 to MED222_13980), *Marinomonas* sp. MED121 (#1: genes MED121_07350 to MED121_07465; #2: MED121_11870 to MED121_11955), *Roseobacter* sp. MED193 (MED193_00960 to MED193_01055).

The type VI secretion system (T6SS) represents another pathway for protein translocation (Mougous *et al*., 2006; Pukatzki *et al*., 2006). The genes of the T6SS constitute a genomic island important for the virulence/antivirulence of a large number of animal and plant pathogens (Das and Chaudhuri, 2003). Searches specific for core conserved T6SS proteins revealed the presence of putative virulence factor homologues in 23 gammaproteobacteria and 12 alphaproteobacteria (Table 1). Proteins of the T6SS operons in the marine bacteria showed amino acid sequence similarities between 29 and 85% to the corresponding T6SS proteins in *Salmonella enterica* ssp. I (the Sci-operon; Folkesson *et al*., 2002). Additionally, the planctomycete *Blastopirellula marina* DSM3645 harbours 12 typical T6SS genes, divided into two genome locations. The T6SS genomic islands carry a core set of genes found in almost all of the examined genomes and several additional genes (Fig. 1). Focusing on this core set of genes, the T6SS island of *Roseobacter* sp. MED193 showed the highest similarity to the *S. enterica* ssp. I T6SS genes, being 29–71% and 46–84% at the nucleotide and amino acid levels respectively (see Table S2). The closest match to the *V. cholerae* O1 T6SS was found in *Marinomonas* sp. MED121 (54–85% identical, 79–92% conserved amino acids). That the closest matches were not found in the phylogenetically closest relatives might be an indication of the T6SS genomic island as a functional unit, which may have been acquired from other species through horizontal gene transfer. Genes encoding proteins known to be exported by the *V. cholerae* T6SS, that is, the Hcp (SciK/M) and VgrG proteins, are found within the operon (Pukatzki *et al*., 2006; 2007), and had predicted homologues in all of the marine T6SS islands identified (Fig. 1). The genes *vasA (sciC*) and *vasK (sciS*), necessary for T6SS-mediated secretion in *V. cholerae* V52 (Pukatzki *et al*., 2006), belonged to the core set of genes found in all the marine T6SS islands, whereas the *vasH* gene, also important for T6SS function in *Aeromonas hydrophila* (Suarez *et al*., 2008), had a homologue only in six marine gammaproteobacteria (*Photobacterium* sp. SKA34, *V. angustum* S14, *Marinobacter* spp. DG893 and ELB17, *Psychromonas* sp. CNPT3 and *Marinomonas* sp. MED121). Taken together, our detection of T6SS in 37 out of 119 marine bacterial genomes, where previously unrecognized, corroborate and extend the findings of Bingle and colleagues (2008) that this secretion system is ‘widespread in nature and not confined to known pathogens’.

### ‘Antifeeding islands’

**Fig. 2:**
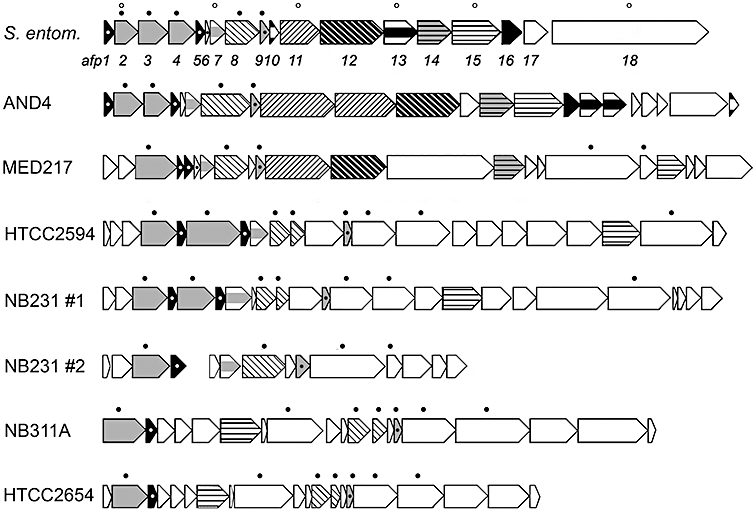
Gene organization of the ‘antifeeding’ prophage-like island in marine bacteria. Genes that are predicted homologues to *S. entomophila*‘antifeeding’ genes are shown with the same patterns as these. Unfilled boxes indicate genes that do not have homologues in *S. entomophila*. Filled black circles above genes indicate genes that show similarity to phage structural genes. Black circles above genes of *S. entomophila* indicate genes that have been shown to be essential for the antifeeding effect of *S. entomophila*. *S. entom*: *Serratia entomophila* A1MO2 AND4, *Vibrio campbellii* AND4; MED217, *Leeuwenhoekiella blandensis* MED217; HTCC2594, *Erythrobacter litoralis* HTCC2594; NB231, *Nitrococcus mobilis* Nb-231 (two loci); NB311A, *Nitrobacter* sp. Nb311A; HTCC2654, *Rhodobacterales bacterium* HTCC2654.

*Serratia entomophila* causes amber disease in larva of the grass grub *Costelytra zealandica*, a plant pest in New Zealand. The pathogenicity of this bacterium, for example, strain A1MO2, derives in part from an ‘antifeeding effect’ that prevents larvae from feeding (Hurst *et al*., 2004). The bacterial genes responsible for the antifeeding effect reside in a locus containing genes that bear similarity to phage structural genes. In the marine bacterium *Vibrio campbellii* AND4 (Fig. 2), a genomic island was detected that contained 16 out of 18 genes in the *S. entomophila* operon constituting the ‘antifeeding island’ (Hurst *et al*., 2004). Amino acid sequence similarities of these putative encoded virulence factors ranged from 43% to 83% (Table S3). Similar genomic islands were found in another 10 marine bacteria (Table 1, Fig. 2), which contained from 8 to 13 of the 18 genes in the *S. entomophila* operon. Notably, the genes *afp*2–4 and *afp*7, identified as essential for the antifeeding effect provided by *S. entomophila*, were present in all the ‘antifeeding island’ copies identified, although some other conserved genes were missing. It was recently shown that the phage-like gene products of the ‘antifeeding operon’ in *Photorhabdus asymbiotica* ATCC43949 form contractile ‘syringe’ structures, and it was suggested that the phage structural proteins could be used to inject toxins into eukaryotic cells (Yang *et al*., 2006).

### *Yersinia* phospholipase-related toxin

The rat flea is the natural vector that spreads *Y. pestis*, the causative agent for plague. In the flea, *Y. pestis* cells growing in the midgut prevent the flea from feeding and increase its attempts to bite, thereby increasing the spread of *Y. pestis*. The *Yersinia*‘murine toxin’ (ymt), a phospholipase D homologue (Hinnebusch *et al*., 2000), is essential for infection of the flea gut. When transferred to *Escherichia coli* the *ymt* gene enables *E. coli*, which normally lacks this gene, to establish a chronic infection of the flea (Hinnebusch *et al*., 2002). Predicted homologues to the *Y. pestis ymt* gene were found in the marine bacteria *Marinomonas* sp. MED121 and *Vibrio* sp. MED222 with a similarity of 64–69% conserved amino acids. Indeed, some bacteria of the Vibrionaceae family are well known to associate to marine arthropods, for example, copepods (Huq *et al*., 1983), possibly indicating a role for the marine *ymt* genes in this context.

### Novel putative PAIs

A frequently used strategy of invasive pathogenic bacteria is to interfere with actin polymerization in the eukaryotic target cell, for example, through interference with Rho GTPases or by directly targeting actin. One such inhibitor of actin polymerization is the mono(ADP-ribosyl)-transferase SpvB from the enteropathogenic bacterium *S. enterica* serovar Dublin, which is known to act both on mammalian and *Acanthamoeba polyphaga* actin polymerization (Tezcan-Merdol *et al*., 2005). The ability to immobilize eukaryotic cells could be useful also for marine bacteria when infecting eukaryotes. Predicted homologues of the *S. enterica* SpvB protein were found in the two Vibrionaceae bacteria *Photobacterium* sp. SKA34 and *Vibrio angustum* S14. In *Photobacterium* sp. SKA34 there were three copies of the *spvB*-like genes (with 41–42% conserved amino acids) in a cluster of six genes, where the other three genes in the operon showed weak similarities to anthrax lethal factor (41% conserved over 360 amino acids), tetanus (53% conserved over 101 amino acids) and botulinum toxins (36% conserved over 328 amino acids) respectively. This cluster is located in a region (∼80 kb, 66 genes) also containing genes with high sequence similarities (i.e. > 40% conserved amino acids) to genes encoding insect toxins, autotransporter adhesins with Ig-domains, a secreted endonuclease, EmpA metallo-protease of the type found in fish-pathogen *V. anguillarum* and a haemolysin/lecithinase produced by *V. cholerae*. Also, other virulence factors, such as putative cytastatin, chitinase, collagenase and penicillin V acyclase genes, as well as a PapC/FimD type usher porin involved in pili biogenesis, were found in this genomic region. *V. angustum* S14 carries a similar genomic region but with some gene rearrangements (Fig. S1), which might be due to that the region is framed and divided by several transposase and phage-type integrase genes. This suggests that the region is part of a mobile genetic element. Similar to many PAIs (Schmidt and Hensel, 2004), also this genomic island is located next to a cluster of tRNA genes.

### Environmental virulence genes

We found a high number of predicted virulence gene homologues in the genomes of cultured marine bacteria, indicating that bacteria carrying such genes can be readily isolated from sea water. However, this observation may not necessarily reflect the occurrence of potential virulence genes among the uncultivated majority of marine bacterioplankton. Therefore, to achieve a first estimate of the prevalence of virulence genes in the ocean we searched the environmental DNA data set generated by the Sorcerer II Global Ocean Sampling Expedition (GOS) including data from 39 marine planktonic samples, a hypersaline lagoon and a lake (Rusch *et al*., 2007). The GOS data set was searched for selected protein sequences from the T4SSs and T6SSs, as well as the (ADP-ribosyl)-transferase of the novel island in *Photobacterium* SKA34 and the ‘antifeeding’ island Afp2. In Table 2 we present the frequencies of these genes normalized to the metagenomic library sizes with marine samples grouped according to high (> 1 μg chl *a* l^−1^) and low (< 1 μg chl *a* l^−1^) chlorophyll *a* concentrations. The investigated virulence genes showed a three to four times higher abundance in productive marine waters (58 genes per 10^9^ bp) and in the hypersaline lagoon (57 genes per 10^9^ bp) compared with open ocean sites (12 genes per 10^9^ bp). The freshwater samples showed an intermediate value (22 genes per 10^9^ bp) with a notable exception in that the T6SS genes were lacking.

**Table 2:** Frequencies of putative virulence genes in the Global Ocean Sampling metagenomic data set.

	Aquatic environment			
Virulence gene homologue	Productive sea water^a^	Oligotrophic sea water^b^	Hypersaline lagoon	Freshwater
T5SS TpsB	42 (66)	5.6 (61)	3.3 (4)	12 (6)
T6SS SciB	1.9 (3)	1.3 (14)	7.5 (9)	0 (0)
T6SS SciS	8.9 (14)	2.9 (31)	41 (50)	0 (0)
‘Antifeeding island’ Afp2	4.5 (7)	1.4 (15)	3.3 (4)	9.7 (5)
(APD-ribosyl)-transferase	0.64 (1)	1.1 (12)	1.7 (2)	0 (0)
Total virulence genes	58 (91)	12.3 (133)	57 (69)	22 (11)
RecA	765 (1203)	512 (5533)	479 (584)	883 (455)
Total virulence genes : RecA (%)	8	2	12	3

a > 1 μg chl *a* l^−1^.

b < 1 μg chl *a* l^−1^.

Marine samples were divided into productive (> 1 μg chl *a* l^−1^, 12 samples) and more oligotrophic (< 1 μg chl *a* l^−1^, 27 samples) regions based on chl *a* data published in Rusch and colleagues (2007). Samples from a hypersaline lagoon (Floreana Island, Ecuador) and from freshwater (Lake Gatun, Panama) were also included. Numbers give gene frequencies per billion bases sequenced. Numbers within parentheses give total number of hits in the CAMERA Global Ocean Sampling Database (http://camera.calit2.net/).

The frequency of the typical single-copy *recA* gene was used to estimate the abundance of the virulence genes as a fraction of the total number of genomes (Table 2; Karlin *et al*., 1995; Rusch *et al*., 2007). From this analysis, we estimate that the investigated putative virulence genes were present in 8–12% of the genomes from the productive marine localities and the hypersaline lagoon, but were less common in more oligotrophic marine localities and in freshwater (2–3%; Table 2). These values may be underestimates due to queries being limited to only a few selected virulence genes or overestimates due to the presence of several virulence genes/copies per bacterial genome. Nevertheless, the GOS subset of randomly sequenced environmental DNA support our finding from whole-genome sequences that virulence gene homologues have a widespread occurrence in marine bacteria, particularly in productive waters with high densities of algae. In this scenario, expression of virulence genes as a means to obtain resources could provide a selective advantage for opportunistic bacteria. In contrast, one would not expect to find homologues of virulence genes in typically oligotrophic marine bacteria like *Pelagibacter ubique* that is characterized by small size and a slow growing single-cell life strategy (Morris *et al*., 2002; Simu and Hagstrom, 2004; Giovannoni *et al*., 2005). Accordingly, we found no predicted homologues of virulence genes in any of the three sequenced strains of *P. ubique* (Table 1 and Table S1).

### Concluding remarks

In light of our findings it is reasonable to question if presence of virulence gene homologues fits with what is known about marine bacteria. Unfortunately, a comprehensive understanding of their life strategies is largely lacking. However, some interesting patterns have emerged. For example, several of the *Roseobacter* clade bacteria appear to be associated with phytoplankton (Allers *et al*., 2007; Moran *et al*., 2007), and their containing T4SSs may provide a means to retrieve resources from these eukaryotes. For fast-growing gammaproteobacteria, such as *Vibrio* and *Alteromonas* species, labile organic carbon substrates, for example, amino acids or glucose, stimulates growth (Eilers *et al*., 2000; Pinhassi and Berman, 2003). Also organic matter released during intense algal blooms stimulates the growth of *V. cholerae* O1 in sea water (Mourino-Pérez *et al*., 2003). At this stage it can only be speculated that the use for specific protein secretion systems and other virulence gene homologues enable, or even is a prerequisite, for a fruitful transition from a free-living lifestyle to life attached to phytoplankton or crustaceans.

We screened for only a subset of known virulence genes and PAIs, and thus our data should represent a conservative estimate of the number of potential virulence genes in marine bacteria. Still, the presence of virulence genes does not necessarily imply that these bacteria are pathogens (e.g. on humans or fish). Even though our sequence analysis shows that marine bacteria have genetic homologues of virulence genes in known pathogens, the function of the expressed proteins may differ between organisms. For example, marine bacteria could use secretion systems and adhesins to attach to each other, other bacteria or to floating particles. However, it is plausible that such traits could also be useful to attach to and exploit compromised, dying or dead single-celled or multicellular eukaryotes. In so doing, virulence genes and PAIs could constitute the genetic tools for marine bacteria to cause harm to algae, and thereby contribute to explaining some of the ample records of such behaviour (Mayali and Azam, 2004). Alternatively, or in addition, ‘virulence’ genes may play an important role in protecting bacteria against protozoan predation (Matz and Kjelleberg, 2005). These authors also proposed that microbial virulence factors associated with human disease could originate from such antipredator defence.

Interestingly, it was recently suggested that host–pathogen interactions should be studied in a wider ecological and evolutionary perspective to better understand the life strategy of pathogenic bacteria (Pallen and Wren, 2007). Functions that have evolved under long time in nature may have been recruited through horizontal gene transfer to perform similar or different functions by more recently emerging pathogenic bacterial species. The precise functions of the putative virulence gene homologues reported here remain unknown until experimental genetics and physiological response experiments can confirm their native function; for example, are they used to support host association, mutualism or commensalism or infection? Whatever the case may be, our analysis show that several marine bacteria contain virulence gene homologues that could potentially be used to attach to, and take advantage of, eukaryotic cells in a manner similar to that used by known pathogens (i.e. as an intrusive way to obtain organic matter and nutrients). Such functions could be important for understanding the ecology and evolution of marine bacteria.

In conclusion, we found a widespread occurrence of virulence gene homologues in a phylogenetically diverse set of genome sequenced marine bacteria. Further, our analysis of environmental DNA suggests that several of these genes are widely distributed in the oceans. Consequently, marine bacteria may constitute a reservoir for virulence genes found in human, animal, plant and insect pathogens as previously suggested for virulence genes in soil bacteria (Casadevall, 2006). From a marine ecology perspective, expression of these genes would indicate that some bacteria infect or even consume live cells. This would result in a previously unrecognized flow of organic matter and nutrients directly from eukaryotes to bacteria (Fig. S2).

## Experimental procedures

### Analyses of genomes of marine bacteria

Whole-genome sequencing was done by the J. Craig Venter Institute (JCVI; sequences and strain information available at https://moore.jcvi.org/moore/) within the Gordon and Betty Moore Foundation Marine Microbiology Initiative (http://www.moore.org/marine-micro.aspx). Open reading frames were predicted and annotated using the annotation pipeline at JCVI, which integrates results from hidden Markov models and blast. Functional prediction was carried out using COG analysis and motif finding. Non-coding RNA prediction was done using tRNA-scanSE and blast. All automatically annotated genes of interest were inspected and verified manually by blast (Altschul *et al*., 1990), COG, PFAM and TIGRFAM analyses. If the annotation was judged to be correct, the analyses were further extended by repeating the analyses on neighbouring genes and by searching for elements (tRNA genes, IS sequences, transposons, phage/plasmid replicons and deviating GC content) indicative of mobile genetic regions. When a putative genomic island had been identified, homologous islands were screened for in other marine bacteria, where in addition to the aforementioned analysis, attention was also paid to gene order and operon structure in homologous genomic islands to verify that the putative island was indeed a conserved genomic unit.

An initial analysis was made on the genome sequences from marine bacterial isolates that we contributed to the Marine Microbiology Initiative and made available to us directly after sequencing. These genomes were: *Neptuniibacter caesariensis* MED92, *Marinomonas* sp. MED121, *Dokdonia* sp. MED134, *Polaribacter* sp. MED152, *Roseobacter* sp. MED193, *Leeuwenhoekiella blandensis* MED217, *Vibrio* sp. MED222, *Reinekea* sp. MED297, *Oceanobacter* sp. RED65, *Photobacterium* sp. SKA34, *Loktanella vestfoldensis* SKA53 and *Sphingomonas* sp. SKA58. In a first step, to find potential virulence gene homologues, we screened the automatic JCVI annotation by searching for virulence and secretion system genes or by looking for signs of defect prophage and IS elements indicating the presence of mobile genetic elements.

We also conducted blastp searches using genes of known virulence factors from animal and human pathogens as query sequences. In particular, we looked for tyrosine kinases, ADP-ribosyltransferases, various known and suspected haemolysins using characterized virulence genes from *S. enterica* (e.g. *spvB*, *hlyA*) and *Y. pestis* (e.g. *ymt*, *yopH*). For each of the virulence gene systems analysed, the original pathogen genomes where the virulence genes are present were included in the screening as positive controls to confirm that the methodology used could detect the relevant genes. Standard blastp was made with blastp 2.2.14 with no filtering, expectancy: 10, word size: 3, scoring matrix: blosum 62, gap costs: opening 11, extension 1. Virulence factor homologues were identified on the criteria of an *E*-value ≤ 10^−6^ and sequence similarity > 30%.

#### Type III secretion system

The T3SSs were located using a combination of PFAMs and TIGRFAMs that target peptides associated with T3SSs and are essential for virulence (references), for example, TIGR02500 (YscD/HrpQ), PF05932 (CesT), TIGR02497 (YscI/HrpB), TIGR02499 (HrpE/YscL) and PFAMs associated with protein exportation, that is, PF01514 (YscJ/FliF), PF00813 (inner membrane P protein FliP), PF00771 (Flagellar/Hr/Invasion Proteins Export Pore), PF01313 (export protein FliQ, family 3) and PF01312 (type III secretion exporter). Bacterial genomes with hits against several of these domains were manually inspected to exclude operon structures containing genes involved in flagellar biosynthesis.

#### Type IV secretion system

The T4SSs were screened for among the marine bacterial genomes using PF04610 (TrbL/VirB6 plasmid conjugal transfer protein) and PF04335 (VirB8 protein) and an *E*-value ≤ 10^−6^. Genomes with positive hits for these PFAMs were manually inspected for presence of the T4SS core conserved protein classes VirB4, VirB6, VirB8, VirB9, VirB10, VirB11, VirD4 (Medini *et al*., 2006). Operon structures with genes for these seven proteins were thus detected using blastp with known VirB proteins from *A. tumefaciens* and PFAM and COG matches. The *vir* gene homologues encoding these proteins were all present in the genomes scored as T4SS positive and presented in Table 1.

#### Type V secretion system

We screened for TPS (or T5bSS) systems using a combination of the PFAMs PF05860 (filamentous haemagglutinin, N-terminal domain), PF08479 (polypeptide transport-associated, ShlB-type) and PF03865 (haemolysin activator HlyB) and an *E*-value ≤ 10^−6^. Positive hits were manually inspected to confirm that the two components of the TPS system were located in adjacent open reading frames.

#### Type VI secretion system

The T6SSs were identified essentially as described by Bingle and colleagues (2008), using the seven T6SS-specific PFAM domains PF05591 (DUF770), PF05936 (DUF876), PF05943 (DUF877), PF05947 (DUF879), PF06996 (DUF1305), PF06761 (ImcF-related) and PF06812 (ImpA, N-terminal domain) and an *E*-value ≤ 10^−6^. Genomes were scored as positive for T6SS based on the presence of these markers combined. Some of the genomes had two or more paralogues of the genes containing these PFAMs.

*Yersinia* murine toxine was searched for among the marine bacterial genomes using blastp with the Ymt amino acid sequence from *Y. pestis* strain CO92 (GenBank Accession Number NP_395420) and an *E*-value ≤ 10^−6^ and sequence identity values > 30%.

In summary, functional annotation was based on: (i) sequence similarity values in blast searches over the entire length of the queried genes (unless specifically stated otherwise in the text), all sequence comparisons were made at the protein level, sometimes complemented by DNA sequence analysis, (ii) classification of proteins within the COG, PFAM or TIGRFAM domain structure libraries (Marchler-Bauer and Bryant, 2004) and (iii) gene arrangement/complement, that is, presence in the same genetic context in the marine bacteria as in known pathogens, that is, within the operon structure of defined PAIs.

### Gene frequencies in the Global Ocean Sampling data

We used blast analysis, using virulence factor sequences from the marine bacterial genomes as queries, to find and identify putative virulence factors in the CAMERA Global Ocean Sampling Database (http://camera.calit2.net/). Proteins from the following organisms were used to search the Global Ocean Sampling data (Rusch *et al*., 2007): T5SS TspB homologue: *Oceanobacter* sp. RED65, T6SS SciB and SciS homologues: *Roseobacter* sp. MED193, ‘Antifeeding island’ Afp2 homologue: *L. blandensis* MED217, *Yersinia* murine toxin homologue: *Vibrio* sp. MED222 (APD-ribosyl)-transferase homologue: *Photobacterium* sp. SKA34. Matches to *Burkholderia* were excluded from the analysis as these sequences may stem from contaminants (Rusch *et al*., 2007).

## Supporting information

**Fig. S1.** Novel PAI in *Photobacterium* sp. SKA34 and *Vibrio angustum* S14. Genes with more than single copies, showing homologies to different classes of virulence genes, are marked in different colours (see legend below figure). The genome regions span from SKA34_08238 to SKA34_08563 and VAS14_15892 to VAS14_16212.

**Fig. S2.** Conceptual model of the carbon flow in the marine pelagic. Grey lines represent release of dissolved organic carbon as the result of several different mechanisms including virus lysis. The red lines depict possible added effects of putative virulence genes on the carbon fluxes in the marine planktonic food web.

**Table S1.** List of genomes included in the study. Genomes marked in boldface contained some/several of the virulence genes or PAIs that were screened for. Genomes were sequenced through the Moore Foundation Initiative in Marine Microbiology.

**Table S2.** Comparison of the proteins in type VI secretion systems of *Salmonella enterica* subsp. Typhimurium (SALTY), *Vibrio cholerae* (V.chol.), *Rhizobium leguminosarum* (RHIZO) and marine bacteria (*Roseobacter* sp. MED193, *Marinomonas* sp. MED121, *Vibrio* sp. MED222, *Reinekea* sp. MED297, *Oceanobacter* sp. RED65, *Photobacterium* sp. SKA34). Standard blastp was made with blastp 2.2.14 with no filtering, expectancy: 10, word size: 3, scoring matrix: blosum 62, gap costs: opening 11, extension 1. Numbers given represent ‘% amino acid identity/% aa similarity/length in aa of matched region’. The conserved domains in the table legends indicate domains detected upon blastp analyses in GenBank.

**Table S3.** Comparison between antifeeding PAIs from *Serratia entomophila* (Afp1–Afp18), *Photorhabdus luminescence* and a number of marine bacteria (see https://research.venterinstitute.org/moore/MultiOrganism?

MULTIORGANISM_SORT_ATTR=SORT_ORGANISM for sequence access). Number in parenthesis represent ‘% amino acid identity/% aa similarity’ as found in standard blastp search without low-complexity filtering and blosum 45 scoring matrix. Proteins were searched for PFAM and COG protein motifs.
