# Supplementary material for: High abundance of virulence gene homologues in marine bacteria
Source: Environ Microbiol. 2009 Jun;11(6):1348–57. doi: 10.1111/j.1462-2920.2008.01861.x (PMC2702493; doi:10.1111/j.1462-2920.2008.01861.x)
Supplement: Supplementary file 1 [file emi0011-1348-SD1.doc]

**
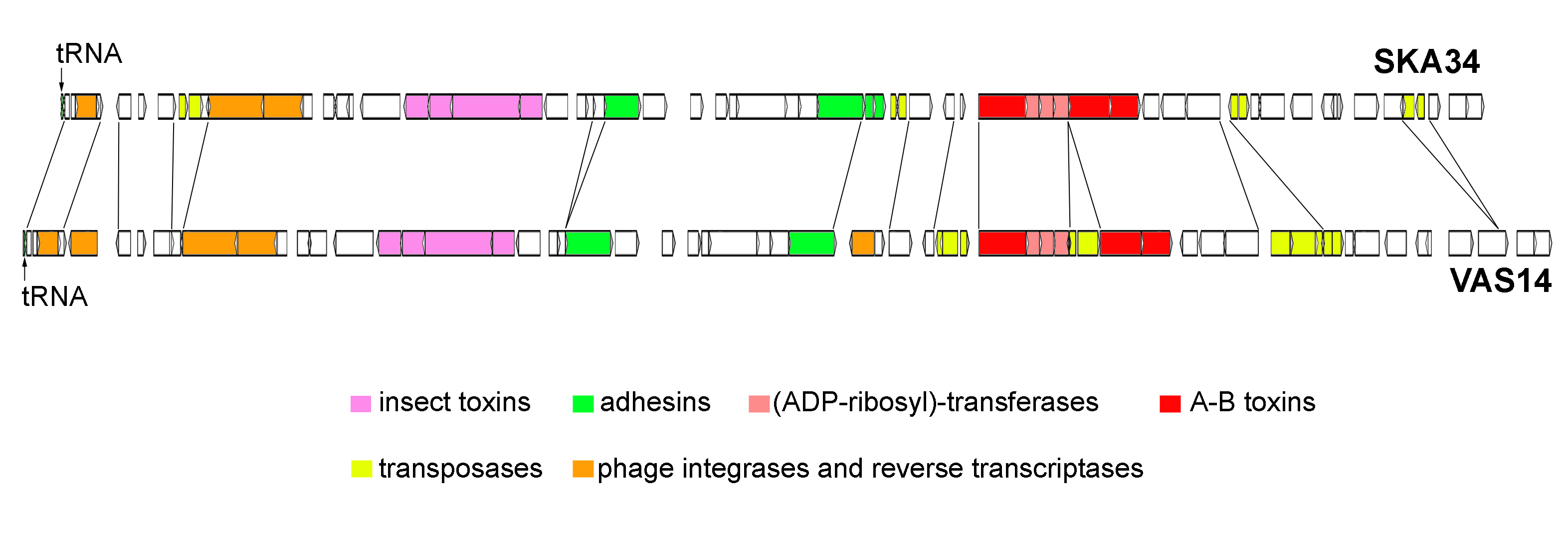
**

**Figure S1**. Novel pathogenicity island in *Photobacterium* sp. SKA34 and *Vibrio angustum* S14. Genes with more than single copies, showing homologies to different classes of virulence genes, are marked in different colors (see legend below figure). The genome regions span from SKA34_08238 to SKA34_08563 and VAS14_15892 to VAS14_16212.

**Figure S2**. Conceptual model of the carbon flow in the marine pelagic. Grey lines represent release of dissolved organic carbon (DOC) as the result of several different mechanisms including virus lysis. The red lines depict possible added effects of putative virulence genes on the carbon fluxes in the marine planktonic foodweb.
