# Supplementary material for: High abundance of virulence gene homologues in marine bacteria
Source: Environ Microbiol. 2009 Jun;11(6):1348–57. doi: 10.1111/j.1462-2920.2008.01861.x (PMC2702493; doi:10.1111/j.1462-2920.2008.01861.x)
Supplement: Supplementary file 2 [file emi0011-1348-SD2.doc]

**Supplementary Table 1.** List of genomes included in the study. Genomes marked in boldface contained some/several of the virulence genes or pathogenicity islands that were screened for. Genomes were sequenced through the Moore Foundation initiative in Marine Microbiology.

| *Aciduliprofundum boonei* T469 |
| --- |
| ***Alcanivorax* sp. DG881** |
| ***Algoriphagus* sp. PR1** |
| **alpha proteobacterium BAL199** |
| **Alteromonadales TW-7** |
| *Alteromonas macleodii* Deep ecotype |
| *Aurantimonas* sp. SI85-9A1 |
| *Bacillus* sp. B14905 |
| *Bacillus* sp. NRRL B-14911 |
| *Bacillus* sp. SG-1 |
| **beta proteobacterium KB13** |
| ***Blastopirellula marina* DSM 3645** |
| ***Brevundimonas* sp. BAL3** |
| *Caminibacter mediatlanticus* TB-2 |
| Campylobacterales bacterium GD 1 |
| *Carboxydibrachium pacificum* str. DSM 12653 |
| *Carnobacterium* sp. AT7 |
| *Congregibacter litoralis* KT71 |
| *Croceibacter atlanticus* HTCC2559 |
| *Dokdonia donghaensis* strain MED134 |
| ***Erythrobacter litoralis* HTCC2594** |
| ***Erythrobacter* sp. NAP1** |
| ***Erythrobacter* sp. SD-21** |
| Flavobacteria bacterium BAL38 |
| Flavobacteria bacterium BBFL7 |
| Flavobacteriales bacterium ALC-1 |
| Flavobacteriales bacterium HTCC2170 |
| ***Fulvimarina pelagi* HTCC2506** |
| gamma proteobacterium HTCC5015 |
| Gammaproteobacterium NOR51-B |
| Gammaproteobacterium NOR5-3 |
| *Hoeflea phototrophica* DFL-43 |
| *Hydrogenivirga* sp. 128-5-R1-1 |
| *Idiomarina baltica* OS145 |
| *Janibacter* sp. HTCC2649 |
| ***Kordia algicida* OT-1** |
| ***Leeuwenhoekiella blandensis* strain MED217** |
| *Lentisphaera araneosa* HTCC2155 |
| ***Limnobacter* sp. MED105** |
| *Loktanella vestfoldensis* SKA53 |
| *marine actinobacterium* PHSC20C1 |
| **Gammaproteobacterium HTCC2080** |
| marine gamma proteobacterium HTCC2143 |
| marine gamma proteobacterium HTCC2148 |
| *Marinitoga piezophila* KA3 |
| ***Marinobacter* sp. DG893** |
| ***Marinobacter* sp. ELB17** |
| ***Marinomonas* sp. MED121** |
| *Mariprofundus ferrooxydans* PV-1 |
| ***Methylophaga* sp. DMS010** |
| **Methylophilales bacterium HTCC2181** |
| ***Microscilla marina ATCC 23134*** |
| ***Moritella* sp. PE36** |
| ***Nitrobacter* sp. Nb-311A** |
| ***Nitrococcus mobilis* Nb-231** |
| *Nitrosococcus oceani* AFC-27 |
| ***Oceanibulbus indolifex* HEL-45** |
| *Oceanicaulis alexandrii* HTCC2633 |
| ***Oceanicola batsensis* HTCC2597** |
| *Oceanicola granulosus* HTCC2516 |
| ***Oceanobacter* sp. RED65** |
| *Oceanospirillum* sp. MED92 |
| *Octadecabacter antarcticus* str. 238 |
| *Octadecabacter antarcticus* str. 307 |
| *Parvularcula bermudensis* HTCC2503 |
| *Pedobacter* sp. BAL39 |
| *Pelagibacter ubique* HTCC1002 |
| *Pelagibacter* HTCC7211 |
| *Pelagibacter* *ubique* HTCC1062 |
| *Phaeobacter gallaeciensis* 2.10 |
| ***Phaeobacter gallaeciensis* BS107** |
| *Photobacterium profundum* 3TCK |
| ***Photobacterium* sp. SKA34** |
| *Planctomyces maris* DSM 8797T |
| ***Plesiocystis pacifica* SIR-1** |
| *Polaribacter dokdonensis* strain MED152 |
| *Polaribacter irgensii* 23-P |
| ***Pseudoalteromonas tunicata* D2** |
| ***Pseudovibrio* sp. JE062** |
| ***Psychromonas* sp. CNPT3** |
| ***Reinekea* sp. MED297** |
| **Rhodobacterales bacterium HTCC2083** |
| Rhodobacterales bacterium HTCC2150 |
| **Rhodobacterales bacterium HTCC2654** |
| **Rhodobacterales bacterium Y4I** |
| **Rhodobacterales KLH11** |
| *Robiginitalea biformata* HTCC2501 |
| ***Roseobacter litoralis* Och 149** |
| *Roseobacter* sp. AzwK-3b |
| ***Roseobacter* sp. CCS2** |
| ***Roseobacter* sp. GAI101** |
| ***Roseobacter* sp. MED193** |
| ***Roseobacter* sp. SK209-2-6** |
| ***Roseovarius* nubinhibens ISM** |
| ***Roseovarius* sp. 217** |
| ***Roseovarius* sp. HTCC2601** |
| ***Roseovarius* sp. TM1035** |
| *Ruegeria* sp. R11 |
| ***Sagittula stellata* E-37** |
| *Shewanella benthica* KT99 |
| ***Sphingomonas* sp. SKA58** |
| ***Stappia aggregata* IAM 12614** |
| *Stappia alexandrii* DFL-11 |
| ***Stenotrophomonas* sp. SKA14** |
| *Sulfitobacter* sp. EE-36 |
| ***Sulfitobacter* sp. NAS-14.1** |
| *Thermococcus* MP |
| *Thermococcus* sp. AM4 |
| unidentified eubacterium SCB49 |
| Verrucomicrobiales bacterium DG1235 |
| ***Vibrio alginolyticus* 12G01** |
| ***Vibrio angustum* S14** |
| ***Vibrio campbellii* AND4** |
| ***Vibrio fischeri* MJ11** |
| *Vibrio parahaemolyticus* 16 |
| ***Vibrio shiloi* AK1** |
| ***Vibrio* sp. MED222** |
| ***Vibrio splendidus* 12B01** |
| **Vibrionales bacterium SWAT-3** |
