# Supplementary material for: High abundance of virulence gene homologues in marine bacteria
Source: Environ Microbiol. 2009 Jun;11(6):1348–57. doi: 10.1111/j.1462-2920.2008.01861.x (PMC2702493; doi:10.1111/j.1462-2920.2008.01861.x)
Supplement: Supplementary file 3 [file emi0011-1348-SD3.doc]

**Supplementary Table 2**. Comparison of the proteins in type 6 secretion systems of *Salmonella enterica* subsp Typhimurium (SALTY), *Vibrio cholerae* (V. chol), *Rhizobium leguminsarum* (RHIZO), and marine bacteria (*Roseobacter* sp MED193, *Marinomonas* sp. MED121, *Vibrio* sp. MED222, *Reinekea* sp. MED297, *Oceanobacter* sp. RED65, *Photobacterieum* sp. SKA34) Standard blastp was made with blastp 2.2.14 with no filtering, expectancy: 10, word size: 3, ccoring matrix: BLOSUM 62, gap costs: opening 11, extention 1. Numbers given represent “% amino acid identity/ % aa similarity/ length in aa of matched region”. The conserved domains in the table legends indicate domains detected upon BLASTP analyses in GenBank.

**Table 2a.** SciA, COG3515, pfam06812

|  | MED193 1055 | MED121_1 7430 | MED121_2 11880 | MED121_2 11870 | MED222  13965 | MED297 18438 | RED65 890 | SKA34 5625 | RHIZO ImpA | V.chol.  VCA119 | V.chol.  VCA121 |
| --- | --- | --- | --- | --- | --- | --- | --- | --- | --- | --- | --- |
| SALTY  SciA | 25/42/367 | 21/38/372 |  |  |  | 21/40/187 |  |  | 24/38/344 |  |  |
| MED193 1055 | - | 21/38/315 |  |  |  |  |  |  | 20/39/350 |  |  |
| MED121_1 7430 |  | - |  |  | 37/53/494 |  | 25/43/132 |  | 24/42/399 |  |  |
| MED121_2 11880 |  |  | - |  |  | 23/42/416 | 24/41/514 | 47/65/480 |  | 49/67/461 |  |
| MED121_2 11870 |  |  |  | - |  |  | 23/49/91 |  |  |  | 31/52/427 |
| MED222  13965 |  |  |  |  | - |  |  |  | 22/41/409 |  |  |
| MED297 18438 |  |  |  |  |  | - | 23/48/498 | 21/28/481 |  | 21/39/411 |  |
| RED65 895 |  |  |  |  |  |  | - | 20/40/533 | 24/42/102 | 23/40/513 |  |
| SKA34 5625 |  |  |  |  |  |  |  | - |  | 44/60/472 |  |
| RHIZO impA |  |  |  |  |  |  |  |  | - |  |  |
| V.chol.  VCA119 |  |  |  |  |  |  |  |  |  | - |  |

**Table 2b.** SciB, DUF1305, pfam06996

|  | MED193 1020 | MED121_1 7395 | MED121_2 11920 | MED222 13930 | MED297 18408 | RED65 830 | SKA34 5680 | RHIZO ImpH | V.chol. VCA0111 | DSM3645 11342 |
| --- | --- | --- | --- | --- | --- | --- | --- | --- | --- | --- |
| SALTY SciB | 35/53/272 |  | 25/46/276 |  | 22/45/260 | 21/39/243 | 25/45/276 | 29/43/264 | 25/45/270 | 30/47/275 |
| MED193 1020 | - |  | 26/46/264 | 19/38/316 | 23/43/261 | 23/41/320 | 26/42/265 | 30/44/287 | 24/42/302 | 31/47/351 |
| MED121_1 7395 |  | - | 20/38/299 | 34/58/317 | 23/40/283 |  | 21/39/300 |  |  | 24/42/333 |
| MED121_2 11920 |  |  | - | 20/38/228 | 27/45/284 | 23/42/317 | 69/81/314 | 24/39/304 | 64/78/315 | 27/46/13 |
| MED222 13930 |  |  |  | - | 20/42/276 | 23/39/296 | 20/38/285 |  | 2/040/264 | 23/43/351 |
| MED297 18408 |  |  |  |  | - | 28/49/143 | 25/41/280 | 25/39/241 | 23/42/299 | 22/37/333 |
| RED65 835 |  |  |  |  |  | - | 24/44/312 |  | 23/42/277 | 27/42/322 |
| SKA34 5680 |  |  |  |  |  |  | - | 25/43/247 | 61/75/341 | 29/47/350 |
| RHIZO ImpH |  |  |  |  |  |  |  | - | 24/43/238 | 32/50/280 |
| V.chol. VCA0111 |  |  |  |  |  |  |  |  |  | 27/46/355 |

**Table 2c.** SciC, DUF879, COG3519

|  | MED193 1025 | MED121_1 7400 | MED121_2 11925 | MED222 13935 | MED297 18413 | RED65 835 | SKA34 5685 | RHIZO ImpG | V.chol. VCA0110 VasA |
| --- | --- | --- | --- | --- | --- | --- | --- | --- | --- |
| SALTY SCIC | 35/53/627 | 24/44/612 | 25/44/629 | 20/41/631 | 25/41/602 | 25/42/623 | 24/43/628 | 31/47/626 | 28/45/638 |
| MED193 1025 | - | 22/42/610 | 28/46/628 | 22/41/603 | 25/44/629 | 24/42/625 | 28/47/628 | 33/50/617 | 30/47/637 |
| MED121_1 7400 |  | - | 24/45/595 | 40/63/593 | 24/42/583 | 23/41/566 | 21/43/582 | 23/42/600 | 22/43/593 |
| MED121_2 11925 |  |  | - | 23/42/586 | 26/47/605 | 28/52/572 | 70/82/588 | 29/48/607 | 68/80/590 |
| MED222 13935 |  |  |  | - | 40/63/593 | 22/41/574 | 24/45/590 | 26/43/601 | 23/42/488 |
| MED297 18413 |  |  |  |  | - | 26/50/577 | 24/46/604 | 25/44/591 | 23/44/593 |
| RED65 840 |  |  |  |  |  | - | 28/53/574 | 25/45/578 | 31/51/578 |
| SKA34 5685 |  |  |  |  |  |  | - | 31/47/609 | 65/80/590 |
| RHIZO ImpG |  |  |  |  |  |  |  | - | 30/49/609 |

**Table 2d.** SciD, COG3518, pfam07025

|  | MED193 1030 | MED121_1 7405 | MED222 13940 | RHIZO ImpF |
| --- | --- | --- | --- | --- |
| SALTY SciD | 42/57/155 |  |  | 31/47/123 |
| MED193 1030 | - |  |  | 30/52/117 |
| MED121_1 7405 |  | - | 38/63/136 | 27/47/119 |
| MED222 13940 |  |  | - |  |

**Table 2e.** VCA0109, COG3518, pfam07025

|  | MED297 18418 | SKA34 5690 | V.chol. VCA0109 |
| --- | --- | --- | --- |
| MED121_2 11930 | 27/50/79 | 66/79/144 | 66/79/145 |
| MED297 18418 | - | 29/49/79 | 27/45/125 |
| SKA34 5690 |  | - | 59/75/144 |

**Table 2f.** SciE, COG4455, pfam07024

|  | MED193 1035 | RHIZO ImpE |
| --- | --- | --- |
| SALTY SciE | 40/55/255 | 29/46/262 |
| MED193 1035 | - | 27/42/248 |

**Table 2g.** SciG, COG0542, pfam00004

|  | MED193 1015 | MED121_1 7390 | MED121_2 11985 | MED222 13925 | MED297 18403 | RED65 780 | SKA34 5640 | V.chol. VCA0116 |
| --- | --- | --- | --- | --- | --- | --- | --- | --- |
| SALTY SciG | 52/68/900 | 43/62/866 | 39/57/911 | 42/61/884 | 39/57/915 | 45/64/871 | 42/58/891 | 41/59/883 |
| MED193 1015 | - | 43/63/907 | 41/58/911 | 43/63/899 | 40/59/926 | 44/62/910 | 41/59/904 | 41/59/909 |
| MED121_1 7390 |  | - | 40/58/894 | 68/80/871 | 40/61/892 | 49/68/869 | 41/60/878 | 41/60/878 |
| MED121_2 11985 |  |  | - | 40/57/897 | 40/59/922 | 40/59/904 | 70/83/898 | 68/80/897 |
| MED222 13925 |  |  |  | - | 40/61/894 | 50/67/869 | 42/60/880 | 40/59/873 |
| MED297 18403 |  |  |  |  | - | 40/59/905 | 40/59/893 | 41/59/887 |
| RED65 780 |  |  |  |  |  | - | 41/61/885 | 41/62/878 |
| SKA34 5640 |  |  |  |  |  |  | - | 71/83/875 |

**Table 2h.** SciH, pfam05591, DUF770

|  | MED193 1000 | MED193 1050 | MED121_1 7420 | MED121_2 11940 | MED222 13955 | MED297 18433 | RED65 885 | RED65 850 | SKA34 5700 | RHIZO ImpB | V.chol. VCA0107 |
| --- | --- | --- | --- | --- | --- | --- | --- | --- | --- | --- | --- |
| SALTY SciH | 61/78/172 | 71/84/159 | 33/52/158 | 36/55/118 | 34/55/158 | 30/53/163 | 31/54/146 | 26/44/145 | 23/51/145 | 38/56/155 | 27/51/118 |
| MED193 1000 | - | 77/88/158 | 36/55/157 | 30/49/153 | 37/56/157 | 31/49/182 | 35/49/137 | 19/41/227 | 28/50/147 | 43/56/169 | 28/50/159 |
| MED193 1050 |  | - | 34/54/168 | 27/48/148 | 37/56/156 | 33/55/145 | 37/53/117 | 28/43/114 | 27/57/113 | 41/56/170 | 32/56/114 |
| MED121_1 7420 |  |  | - | 34/49/167 | 85/91/154 | 34/51/152 | 33/54/141 |  | 32/50/140 | 47/69/148 | 34/51/141 |
| MED121_2 11940 |  |  |  | - | 30/49/166 | 31/53/166 | 51/70/154 | 28/52/114 | 72/87/165 | 34/54/150 | 71/85/167 |
| MED222 13955 |  |  |  |  | - | 33/52/157 | 35/54/153 | 26/46/130 | 36/56/149 | 49/71/160 | 33/50/151 |
| MED297 18433 |  |  |  |  |  | - | 40/60/148 | 20/51/149 | 32/56/159 | 35/52/165 | 35/53/160 |
| RED65 855 |  |  |  |  |  |  | - | 32/52/114 | 46/71/156 | 33/56/151 | 46/68/156 |
| RED850  850 |  |  |  |  |  |  |  | - | 28/54/114 |  | 27/49/114 |
| SKA34  5700 |  |  |  |  |  |  |  |  | - | 33/51/149 | 72/86/165 |
| RHIZO ImpB |  |  |  |  |  |  |  |  |  | - | 38/52/150 |

**Table 2i.** SciI, DUF877

|  | MED193 1000 | MED193 1045 | MED121_1 7410 | MED121_1 7415 | MED121_2 11935 | MED222 13945 | MED222 13950 | MED297 18428 | RED65 880 | RED65 850 | SKA34 5695 | RHIZO ImpC | RHIZO ImpD | V.chol. VCA0108 |
| --- | --- | --- | --- | --- | --- | --- | --- | --- | --- | --- | --- | --- | --- | --- |
| SALTY SciI | 31/51/256 | 66/79/500 | 27/46/436 | 42/61/494 | 41/61/457 | 23/43/481 | 42/61/495 | 40/59/424 | 39/59/457 | 30/52/462 | 39/58/456 | 49/69/449 | 35/52/440 | 41/60/457 |
| MED193 1000 | - | 29/48/296 | 26/42/223 | 27/43/205 | 25/42/305 | 21/36/225 | 23/40/291 | 27/45/288 | 26/44/292 | 21/41/272 | 29/45/305 | 27/45/296 | 28/43/232 | 27/43/305 |
| MED193 1045 |  | - | 29/46/425 | 39/59/495 | 40/61/430 | 26/45/477 | 39/60/491 | 38/59/458 | 37/59/432 | 33/53/413 | 36/57/472 | 48/66/499 | 33/52/440 | 41/61/430 |
| MED121_1 7410 |  |  | - | 26/45/446 | 24/47/439 | 49/69/461 | 24/43/461 | 26/46/426 | 27/45/416 | 25/44/408 | 24/46/451 | 28/47/428 | 30/47/427 | 26/47/426 |
| MED121_1 7415 |  |  |  | - | 32/53/477 | 24/42/445 | 80/90/492 | 36/57/440 | 36/56/464 | 29/52/404 | 33/55/464 | 49/68/474 | 30/50/454 | 34/54/464 |
| MED121_2 11935 |  |  |  |  | - | 25/43/439 | 32/53/497 | 46/67/447 | 56/76/490 | 38/60/436 | 78/90/486 | 39/57/484 | 33/53/423 | 85/92/490 |
| MED222 13945 |  |  |  |  |  | - | 24/42/468 | 25/45/434 | 24/43/421 | 24/43/401 | 26/43/438 | 26/44/472 | 27/45/421 | 25/43/450 |
| MED222 13950 |  |  |  |  |  |  | - | 35/57/431 | 35/54/500 | 28/51/450 | 34/53/492 | 49/67/493 | 32/51/443 | 33/53/502 |
| MED297 18428 |  |  |  |  |  |  |  | - | 43/68/448 | 33/57/402 | 47/68/436 | 39/60/428 | 32/53/432 | 42/63/508 |
| RED65 890 |  |  |  |  |  |  |  |  | - | 39/59/433 | 56/78/471 | 35/57/491 | 30/50/436 | 57/77/488 |
| RED65 850 |  |  |  |  |  |  |  |  |  | - | 38/61/418 | 31/50/466 | 28/45/422 | 38/60/418 |
| SKA34 5695 |  |  |  |  |  |  |  |  |  |  | - | 37/55/500 | 32/51/423 | 82/91/489 |
| RHIZO ImpC |  |  |  |  |  |  |  |  |  |  |  | - | 35/54/432 | 38/57/485 |
| RHIZO ImpD |  |  |  |  |  |  |  |  |  |  |  |  | - | 33/52/423 |

**Table 2j.** SciK, SciM, hcp, pfam05638, DUF796

|  | SALTY SciM | MED193 1040 | MED121_1 7425 | MED121_2 11945 | MED222 13960 | MED297 18423 | RED65 875 | RED65 845 | SKA34 5710 | V.chol.  VCA0017 Hcp |
| --- | --- | --- | --- | --- | --- | --- | --- | --- | --- | --- |
| SALTY SciK | 93/98/161 | 28/48/153 |  |  |  | 31/54/51 |  |  |  |  |
| SALTY SciM | - | 27/48/153 |  |  |  | 31/54/51 |  |  |  |  |
| MED193 1040 |  | - | 24/43/147 |  | 24/43/145 |  |  |  |  |  |
| MED121_1 7425 |  |  | -- |  | 61/80/173 |  |  |  |  |  |
| MED121_2 11945 |  |  |  | - |  | 31/49/166 | 39/59/163 | 31/48/168 | 80/95/172 | 78/91/172 |
| MED222 13960 |  |  |  |  | - |  |  |  |  |  |
| MED297 18423 |  |  |  |  |  | - | 30/49/163 | 34/58/158 | 30/48/166 | 34/51/139 |
| RED65 875 |  |  |  |  |  |  | - | 31/52/168 | 39/60/163 | 44/64/163 |
| RED65 845 |  |  |  |  |  |  |  | - | 30/51/168 | 33/50/166 |
| SKA34 5710 |  |  |  |  |  |  |  |  | - | 75/88/172 |

**Table 2k.** VrgS, DUF586, pfam04524, COG3501

|  | MED193 995 | MED121_1 7445 | MED121_2 11955 | MED121_2 12005 | MED222 13980 | MED297 18393 | MED297 18398 | RED65 805 | SKA34 5715 | V.chol. VCA0018 VgrG-2 | V.chol. VCA0123 |
| --- | --- | --- | --- | --- | --- | --- | --- | --- | --- | --- | --- |
| SALTY VrgS | 35/54/505 | 25/43/526 | 30/48/570 | 30/48/632 | 27/44/571 | 25/41/208 | 21/42/507 | 21/41/472 | 33/50/498 | 30/48/587 | 28/48/541 |
| MED193 995 | - | 26/44/726 | 27/46/648 | 28/44/657 | 26/44/692 |  | 22/40/643 | 24750/150 | 31/46/647 | 27/43/637 | 28/44/651 |
| MED121_1 7445 |  | - | 25/45/620 | 27/44/640 | 40/58/688 | 24/46/125 | 23/42/610 | 22/41/333 | 26/45/622 | 28/47/567 | 25/44/617 |
| MED121_2 11955 |  |  | - | 60/74/617 | 27/45/546 | 28/46/179 | 22/41/656 | 23/42/455 | 55/71/617 | 52/69/620 | 50/69/622 |
| MED121_2 12005 |  |  |  | - | 26/44/597 | 30/46/183 | 22/37/661 | 23/41/533 | 60/74/675 | 57/71/643 | 54/69/674 |
| MED222 13980 |  |  |  |  | - |  | 23/39/598 | 22/40/413 | 26/47/639 | 29/46/576 | 28/45/552 |
| MED297 18393 |  |  |  |  |  | - | 26/45/593 | 24/41/105 | 27/40/165 |  |  |
| MED297 18398 |  |  |  |  |  |  | - | 22/39/445 | 24/38/577 | 24/43/673 | 23/42/623 |
| RED65 805 |  |  |  |  |  |  |  | - | 23/39/443 | 23/41/387 | 23/40/471 |
| SKA34 5715 |  |  |  |  |  |  |  |  | - | 57/71/681 | 57/72/620 |
| V.chol. VCA0018 |  |  |  |  |  |  |  |  |  | - | 70/83/665 |

**Table 2l.** MED193_00990**,**

|  | MED121_1 7440 | MED222 13975 |
| --- | --- | --- |
| MED193 990 | 24/39/164 | 30/45/152 |
| MED121_1 7440 | - | 41/58/185 |

**Table 2m.** Rhs1, MED193_00985, COG4104, PAAR

|  | MED193 985 | MED121_1 7435 | MED121_2 11950 | MED222 13970 | V.chol. VCA0105 |
| --- | --- | --- | --- | --- | --- |
| SALTY Rhs1 | 41/53/39 | 43/48/39 |  | 41/53/39 |  |
| MED193 985 | - | 46/55/95 |  | 50/62/95 | 41/53/78 |
| MED121_1 7435 |  | - |  | 71/80/99 |  |
| MED121_2 11950 |  |  | - |  | 65/71/94 |
| MED222 13970 |  |  |  | - |  |

**Table 2n.** ImpI, COG3456, pfam00498, FHA

|  | MED121_1 7380 | MED121_2 11915 | MED222 13905 | SKA34 5675 | RHIZO ImpI | V.chol. VCA0112 |  |
| --- | --- | --- | --- | --- | --- | --- | --- |
| MED193 980 | 33/51/87 | 22/41/192 | 33/47/121 | 22/45/184 |  | 25/42/216 |  |
| MED121_1 7380 | - |  | 32/52/331 |  | 21/38/355 | 24/41/207 |  |
| MED121_2 11915 |  | - | 29/50/116 | 47/62/500 |  | 42/61/513 |  |
| MED222 13905 |  |  | - |  | 32/58/67 | 27/38/192 |  |
| SKA34 5675 |  |  |  | - |  | 45/63/477 |  |
| RHIZO ImpI |  |  |  |  | - | 25/43/128 |  |
|  |  |  |  |  |  | - |  |

**Table 2o.** SciN, COG3521

|  | MED121_1 7375 | MED121_2 11910 | MED222 13900 | MED297 18463 | SKA34 5660 | V. chol. VCA0113 |
| --- | --- | --- | --- | --- | --- | --- |
| SALTY SciN | 24/47/145 | 32/57/125 | 28/50/128 | 31/47/119 | 30/55/125 | 33/57/128 |
| MED121_1 7375 | - |  | 33/57/162 | 23/43/130 | 22/50/154 |  |
| MED121_2 11910 |  | - | 23/52/125 |  | 58/77/146 | 59/78/149 |
| MED222 13900 |  |  | - | 30/53/88 | 25/52/123 | 26/52/125 |
| MED297 18463 |  |  |  | - | 23/47/141 | 17/48/137 |
| SKA34 5660 |  |  |  |  | - | 56/75/151 |

**Table 2p.** SciO, pfam05936, DUF876

|  | MED193 975 | MED121_1 7370 (N) | MED121_1 7365 (C) | MED121_2 11905 | MED222 13895 | MED297 18458 | RED65 865 | SKA34 5655 | RHIZO ImpJ | V.chol. VCA0114 |
| --- | --- | --- | --- | --- | --- | --- | --- | --- | --- | --- |
| SALTY SciO | 33/53/447 | 30/48/239 | 29/48/113 | 33/57/451 | 25/47/448 | 23/43/450 | 25/40/459 | 33/56/447 | 34/55/449 | 34/55/449 |
| MED193 975 | - | 33/51/227 | 27/47/179 | 37/60/446 | 30/51/450 | 23/40/452 | 23/41/460 | 39/59/446 | 46/65/446 | 40/60/442 |
| MED121_1 7370 |  | - |  | 30/53/242 | 48/72/235 | 26/46/219 | 23/44/217 | 30/53/220 | 33/51/254 | 32/53/219 |
| MED121_1 7365 |  |  | - | 24/44/190 | 44/65/195 | 25/53/80 | 22/38/197 | 27/44/198 | 30/50/199 | 27/45/146 |
| MED121_2 11905 |  |  |  | - | 29/50/454 | 23/44/447 | 25/43/467 | 72/87/445 | 39/59/446 | 75/88/444 |
| MED222 13895 |  |  |  |  | - | 22/44/429 | 24/43/470 | 30/50/454 | 30/50/450 | 29/49/452 |
| MED297 18458 |  |  |  |  |  | - | 21/40/476 | 22/44/453 | 26/43/452 | 22/44/443 |
| RED65 870 |  |  |  |  |  |  | - | 24/41/450 | 24/40/472 | 24/43/469 |
| SKA34 5655 |  |  |  |  |  |  |  | - | 40/60/446 | 71/84/445 |
| RHIZO ImpJ |  |  |  |  |  |  |  |  | - | 38/58/446 |

**Table 2q**. SciP, COG3455

|  | MED193 970 | MED121_1 7360 | MED121_2 11900 | MED222 13890 | MED297 18453 | RED65 860 | SKA34 5650( N) | SKA34 5645 (C) | RHIZO ImpK | V. chol VCA0115 |
| --- | --- | --- | --- | --- | --- | --- | --- | --- | --- | --- |
| SALTY SciP | 29/46/394 |  | 32/46/229 | 28/47/94 | 20/44/165 | 25/49/106 |  | 28/50/150 | 26/43/400 | 30/47/224 |
| MED193 970 | - | 25/46/167 | 33/50/222 | 18/47/133 | 22/45/214 | 29/52/97 |  | 29/53/150 | 33/53/401 | 34/53/224 |
| MED121_1 7360 |  | - | 25/54/171 | 33/54/396 | 25/43/162 | 83/51/117 |  | 26/57/92 | 24/45/151 | 22/47/181 |
| MED121_2 11900 |  |  | - | 29/53/133 | 25/43/145 | 26/41/214 | 71/86/91 | 59/76/154 | 29/46/229 | 63/79/247 |
| MED222 13890 |  |  |  | - | 26/45/131 | 29/45/125 |  | 27/44/127 |  | 27/51/154 |
| MED297 18453 |  |  |  |  | - | 36/56/74 |  | 19/40/134 | 26/44/136 | 22/44/177 |
| RED65 860 |  |  |  |  |  | - |  | 25/50/63 |  | 27/50/87 |
| SKA34 5650 |  |  |  |  |  |  | - |  |  | 63/83/92 |
| SKA34 5645 |  |  |  |  |  |  |  | - | 29/48/141 | 57/75/152 |
| RHIZO ImpK |  |  |  |  |  |  |  |  | - | 30/49/161 |

**Table 2r.** VCA0117, COG3829, pfam00158

|  | SKA34 5635 | V.chol. VCA0117 |
| --- | --- | --- |
| MED121_2 11890 | 48/66/515 | 47/66/527 |
| SKA34 5635 | - | 45/65/528 |

PCNPT3_09963, VAS14_12714,

**Table 2s.** VCA0118

|  | SKA34 5630 | V. chol. VCA0118 |
| --- | --- | --- |
| MED121_2 11885 | 44/61/193 | 36/59/195 |
| SKA34 5630 | - | 34/56/225 |

VAS14_12709, PCNPT3_09958

**Table 2t**. SciS, pfam06761, COG3523, IcmF, DUF1215

|  | MED193 965 | MED121_1 7355 | MED121_2 11875 | MED222 13885 | MED297 18448 | RED65 855 | SKA34 5620 | RHIZO ImpL | V.chol. VCA0120 VasK |
| --- | --- | --- | --- | --- | --- | --- | --- | --- | --- |
| SALTY SciS | 30/49/1165 | 21/37/1029 | 24/41/1244 | 21/39/1050 | 24/40/1211 | 33/47/318 | 24/40/1261 | 28/45/1253 | 25/41/1255 |
| MED193 965 | - | 23/41/1056 | 26/45/1220 | 21/38/1018 | 24/42/1264 | 30/47/337 | 26/46/1225 | 32/51/1181 | 25/46/1223 |
| MED121_1 7355 |  | - | 21/39/1102 | 30/52/1127 | 22/41/561 | 23/43/182 | 22/40/1089 | 21/38/1094 | 20/39/1188 |
| MED121_2 11875 |  |  | - | 21/39/1170 | 23/41/1235 | 25/47/266 | 59/79/1161 | 24/42/1192 | 62/81/1181 |
| MED222 13885 |  |  |  | - | 21/39/1219 | 19/40/261 | 21/40/1081 | 21/38/1113 | 23/40/1082 |
| MED297 18448 |  |  |  |  | - | 32/51/362 | 22/41/1242 | 23/40/1255 | 24/41/1246 |
| RED65 855 |  |  |  |  |  | - | 24/48/266 | 27/44/505 | 23/46/266 |
| SKA34 5620 |  |  |  |  |  |  | - | 24/40/1185 | 58/79/1162 |
| RHIZO ImpL |  |  |  |  |  |  |  | - | 24/40/1179 |

NB231_12219, V12G01_07523, VAS14_12699, PCNPT3_09948, V12G01_01470, V12B01_14811, PTD2_02706,

**Table 2u.** SciT, COG3913/COG0631 (only ImpM contains both domains)

|  | MED193 960 | MED193 955 | RHIZO ImpM |
| --- | --- | --- | --- |
| SALTY SciT | 27/42/112 |  |  |
| MED193 960 | - |  | 31/45/142 |
| MED193 955 |  | - | 25/43/232 |

NB231_12224, V12G01_07528, B14911_06291,

**MED121 7350** pfam00069, COG0515, S_TKc

MED121 7350 <-> MED222 13880: 30/48/178

**MED121_07455**

MED121_1 7455 <-> MED222 13920:27/47/703

**MED121_0745, DUF534, COG2984**

MED121_1 7450 <-> MED222 13915: 27/50/337

**MED121_07385, pfam00497**

MED121_1 7385 <--> MED222 13910: 43/64/206
