# Supplementary material for: High abundance of virulence gene homologues in marine bacteria
Source: Environ Microbiol. 2009 Jun;11(6):1348–57. doi: 10.1111/j.1462-2920.2008.01861.x (PMC2702493; doi:10.1111/j.1462-2920.2008.01861.x)
Supplement: Supplementary file 4 [file emi0011-1348-SD4.doc]

**Supplementary Table 2.** Comparison between antifeeding pathogenicity islands from *Serratia entomophila* (Afp1-Afp18)*, Photorhabdus luminescence* and a number of marine bacteria (see https://research.venterinstitute.org/moore/MultiOrganism?MULTIORGANISM_SORT_ATTR=SORT_ORGANISM for sequence access). Number in parenthesis represent (% amino acid identity/% aa similarity) as found in standard blastp search without low complexity filtering and BLOSUM45 scoring matrix. Proteins where searched for Pfam protein motifs at http://pfam.sanger.ac.uk/ and COG protein motifs athttp://stdgen.northwestern.edu/cgi-bin/analyze.cgi?cogs=cogs&dbname=cpneu&gene_id=CPn0007

| COG #, pfam # | *S. ento-mophila* | P. luminescence | *Vibrio campbelli* AND4 | *L. blandensis* MED217 | *N. mobilis* Nb-231 | Rhodo-bacterales HTCC2654 | *E. litoralis* HTCC2594 | *Nitrobacter* sp.  Nb-311A |
| --- | --- | --- | --- | --- | --- | --- | --- | --- |
|  | Afp18 |  |  |  |  |  |  |  |
|  | Afp17 |  |  |  |  |  |  |  |
| Pfam-B_26839, Pfam-B_20106 | Afp16 | orf6 () | 5329 (48/67) |  |  |  |  |  |
| COG0464: AAA+ ATPase ; PF00004 AAA  Pfam-B_14783 | Afp15 | orf5 (72/86)  orf4 (40/57) orf26 (42/58) | 05334 (44/61) | 08560 (45/66) | 6431 (46/63) | 6864 (41/59) | 9370 (40/58) | 1644 (32/47) |
| Pfam-B_8525 | Afp14 | orf3  (38/57)  orf25 (37/55) | 05339 (30/50) | 08585 (23/49) |  |  |  |  |
| Pfam-B_8369; Pfam-B_55641 | Afp13 | orf2 (40/52) orf24 (43/59) | 5319,  (58/72,29 aa) 5324, (28/43)  (5344 med 23 aa)  5349  (51/66 26 aa) 5409 (47/57 41 aa) |  |  |  |  |  |
| Pfam-B_16723 | Afp12 | orf23  (46/62) | 5354 (27/46) | 08595 (24/40) |  |  |  |  |
|  |  |  |  |  |  |  |  |  |
| Pfam-B_29546; Pfam-B_12105 | Afp11 | orf22 (42/58) | 5364 (31/48)  5359 (30/49) | 08600 (23/45) |  |  |  |  |
| Pfam-B_23597: | Afp10 | Orf21  (47/57) | 5294 (45/58) |  |  |  |  |  |
| COG3628 Phage baseplate assembly; protein W  PF04965: Gene 25-like lysozyme | Afp9 | Orf20  (44/63) | 5369 (39/62) | 08605 (37/58) | 6451  (30/57)  3480  (37/54) | 6824  (27/43) | 9330  (25/48) | 1604  (30/50) |
| COG3501  vgrG  PF04524: DUF586 | Afp8 | Orf19  (37/58) | 5374 (29/51) | 08615  (23/45) | 6461  (27/42)  6466  3490  (25/40) | 6834 | 9315  9320  (35/49) | 1614 |
| PF03187: Corona nucleocapsid I  Pfam-B_12054 | Afp7 | orf18  (65/79) | 5379 (41/65) | 08620 (23/43) | 6476  (25/40) |  | 9305  (26/49) |  |
| Pfam-B_30683 | Afp6 | orf19 (50/64) |  | 08625 |  |  |  |  |
| pfam09540: hypothetical phage tail | Afp5 | Orf17  (71/83)  Orf13 (22/43) | 5389 (63/80) | 08630  (32/47) | 6481 (27/48)  3505 (28/45)  6491  (28/47) | 6884 (23/46) | 9300 (23/46) | 1664 (24/42) |
| COG3497  pfam04984 | Afp4 | Orf16 (45/61)  Orf15 (41/60)  Orf14 (33/53) | 5394 (33/49) | 8640 (35/56) | 3510  (25/40)  6496 (33/51)  6486  (27/42) |  | 9285 (30/54)  9295 (27/42) | 1669  (29/48) |
| COG3497  pfam04984 | Afp3 | Afp4 (36/59) Orf15 (47/66)  Orf14 (59/77)  Orf16 (36/57) |  | 8640 (43/58) | 6496 (44/64)  3510 (42/62)  6486 (?/?) | 6889 (37/58) | 9285 (43/62)  9295 (28/48) | 1669 (36/58) |
| COG3497  pfam04984: Phage_sheath_1 | Afp2 | Afp3 (63/83)  Afp4 (40/60)  Orf14 (52/68)  Orf15 (64/83)  Orf16 (38/68) | 5399 (43/59) | 8640  (49/67) | 3510  (28/46)  6496 (43/62)  6486 (29/48) | 6889 (35/56) | 9285 (37/58)  9295 (28/49) | 1669 (37/58) |
| pfam09540: CHP2241_phage tail protein | Afp1 | Afp5 (22/42)  Orf13 (73/88) | 5404 (62/83) | 8635 (38/58) | 6491 (31/50)  6481  (21/46)  3505 (24/51) | 6884 | 9300 (24/43)  9290 (23/49) | 1664 (28/48) |
| PF00959: Phage_lysozyme | Mur1 |  |  |  |  |  |  |  |
| PF09081: DUF1921 | Hol1 |  |  |  |  |  |  |  |
| PF03245: Phage_lysis | Enp1 |  |  |  |  |  |  |  |
| PF05106: Phage_holin_3 | AnfA2 |  |  |  |  |  |  |  |
| COG2050: transcr antiterminator  PF02357: NusG | AnfA1 |  | 5414 |  |  |  |  |  |

**Supplementary Table 2, continued**

| COG #, pfam # | *S. entomophila* | Rhodobacterales KLH11 | *R. litoralis* Och 149 | *M. marina* ATCC 23134 | *Algoriphagus* sp. PR1 | *K. algicida* OT-1 | *P. pacifica* SIR-1 |
| --- | --- | --- | --- | --- | --- | --- | --- |
|  | Afp18 |  |  |  |  |  |  |
|  | Afp17 |  |  |  |  |  |  |
| Pfam-B_26839, Pfam-B_20106 | Afp16 |  |  |  |  |  |  |
| COG0464: AAA+ ATPase ; PF00004 AAA  Pfam-B_14783 | Afp15 | 3456 (45/66/249) | 7699 (43/63/257) | 1851 (41/63/249) | 12680 (38/59/262) | 18517  (40/60/256) | 25241 (39/59/284) |
| Pfam-B_8525 | Afp14 |  | 7694 (28/42/66) | 6079 (24/39/141)  1850  (29/48/161) | 12700 (36/58/55) | 18537  (25/43/202) | 25251-  25246  (28/41/139)  sekvensskarv med fel |
| Pfam-B_8369; Pfam-B_55641 | Afp13 |  |  |  |  |  |  |
| Pfam-B_16723 | Afp12 | 3627 | 7689 (38/55/103) | 6078 (21/40/461)  1849 (24/39/630) | 12710 (22/43/464) | 18542  (22/40/477) | 25256 (23/37/652) |
|  |  |  | 7684 | 6090 |  |  | 25261 |
| Pfam-B_29546; Pfam-B_12105 | Afp11 |  | 7674 (24/41/263) | 6089 (23/42/378)  1845 (23/44/384) | 12715 (23/45/359) | 18547  (20/39/399) | 25266 (25/44/394) |
| Pfam-B_23597: | Afp10 |  |  | 1844  (35/54/131) |  |  | 25271  (32/48/113) |
| COG3628 Phage baseplate assembly; protein W  PF04965: Gene 25-like lysozyme | Afp9 | 3593 (34/57/82) | 7669 (33/50/126) | 6088 (35/48/103)  1843  (34/56/112) | 12720 (41/57/85) | 18552 (29/49/89) | 25276 (38/55/101) |
| COG3501  vgrG  PF04524: DUF586 | Afp8 | 3367  3545  (29/45/200) | 7654 (25/46/533) | 6086 (21/39/459)  1841 (25/45/553) | 12730 (25/44/546) | 18562 (24/44/554) | 25286 (25/45/243) |
| PF03187: Corona nucleocapsid I  Pfam-B_12054 | Afp7 | 3438 (22/41/133) | 7649  (26/48/140) | 6076 (25/41/155)  1840  (36/59/52) | 12735 (24/70/177) | 18567 (22/43/170) | 25291  (33/46/166) |
| Pfam-B_30683 | Afp6 |  |  |  | 12740 |  |  |
| pfam09540: hypothetical phage tail | Afp5 | 3349 (28/49/115) | 7639  (31/51/144) | 6083 (28/50/135)  6084 (29/52/128)  1836 (23/44/119)  1837 (28/28/123)  1838 (37/53/141) | 12745 (45/68/144) | 18582 (25/51/122)  18577 (29/49/134) | 25311 (25/49/127)  25301 (34/54/146) |
| COG3497  pfam04984 | Afp4 | 3734 (31/51/188) | 7629 (41/59/182) | 6082 (37/55/193) | 12755 (37/56/196) | 18587 (33/52/189) | 25316 (36/54/212) |
| COG3497  pfam04984 | Afp3 | 3734 (46/63/180) | 7629 (38/54/262) | 6082 (45/59/204)  1835  (49/68/190) | 12755 (50/69/194) | 18587 (43/60/186) | 25316 (44/65/228) |
| COG3497  pfam04984: Phage_sheath_1 | Afp2 | 3734 (41/55/208) | 7629 (44/62/189)  7639  (33/50/116) | 6082 (40/60/189) | 12755 (49/67/189) | 18587 (42/59/192) | 25316 (50/70/190) |
| pfam09540: CHP2241_phage tail protein | Afp1 | 3577 | 7634 (43/61/151) | 6083 (34/54/151)  6084 (23/42/125)  1836 (41/61/155)  1837 (35/54/153)  1838 (22/42/140) | 12750 (38/57/141) | 18582 (33/56/150)  18577 (24/45/134) | 25311 (40/58/155)  25306 (29/52/142) |
| PF00959: Phage_lysozyme | Mur1 |  |  |  |  |  |  |
| PF09081: DUF1921 | Hol1 |  |  |  |  |  |  |
| PF03245: Phage_lysis | Enp1 |  |  |  |  |  |  |
| PF05106: Phage_holin_3 | AnfA2 |  |  |  |  |  |  |
| COG2050: transcr antiterminator  PF02357: NusG | AnfA1 |  |  |  |  |  |  |

http://pfam.sanger.ac.uk/

http://stdgen.northwestern.edu/cgi-bin/analyze.cgi?cogs=cogs&dbname=cpneu&gene_id=CPn0007
